# Etude longitudinale de la névralgie cervico-brachiale dans le service de neurologie du CHU Gabriel Touré, Bamako (Mali)

**DOI:** 10.11604/pamj.2013.16.46.3093

**Published:** 2013-10-10

**Authors:** Youssoufa Maiga, Amina Ahmed Fara, Youssouf Sogoba, Djibo Diango, Sara Diakite, Mohamed Diallo, Hadiza Ak, Gangaly Diallo, Hamar Alassane Traore

**Affiliations:** 1 Service de Neurologie CHU Gabriel Touré, BP 267, Bamako, Mali; 2 Service de Neurochirurgie, CHU Gabriel Touré, BP 267, Bamako, Mali; 3 Service d'Anesthésie Réanimation, CHU Gabriel Touré, BP 267, Bamako, Mali; 4 Service de Radiologie Hôpital Gabriel Touré, CHU Gabriel Touré, BP 267, Bamako, Mali; 5 Service de Médecine interne CHU Point G, Bamako, Mali; 6 Département de chirurgie du CHU Gabriel Touré, Bamako, Mali

**Keywords:** Névralgie Cervico-Brachiale, douleur neuropathique, DN4, Mali, Cervico-brachial neuralgia, neuropathic pain, DN4, Mali

## Abstract

**Introduction:**

La Névralgie Cervico-Brachiale (NCB) est une pathologie relativement fréquente dans la pratique courante. Elle est pourvoyeuse d'importants coûts médicaux et socio-économiques. Peu de données existent sur la NCB en Afrique.

**Méthodes:**

Il s'agit d'une étude longitudinale, descriptive et prospective qui s'est déroulée du 1er novembre 2009 au 30 Août 2010 au CHU Gabriel Touré de Bamako, Mali. Elle a pour objectif d’étudier les caractéristiques épidémiologiques et cliniques de la NCB. Le diagnostic à été strictement clinique, et la DN4 a permis de déterminer les caractéristiques de cette névralgie. L'intensité de la douleur a été évaluée par l’échelle verbale simple (EVS). L’échelle concis de la douleur et l’échelle HAD ont permis d’étudier l'impact de la douleur sur la qualité de vie des patients.

**Résultats:**

La fréquence de la NCB est de 10,9%. Les ménagères sont les plus touchées, 21(40,4%). L’âge moyen des patients est de 48 ±7 ans. La tranche d’âge de 50-59 ans représente la classe modale. La douleur est à prédominance nocturne chez 75,0% des patients. Les décharges électriques sont la caractéristique principale soit 48,1% des patients et 57,7% malades présentent une douleur intense. Sur le plan topographique, la racine C7, est la plus atteinte soit 50,0%. Sur la qualité de la vie, 44,2% des malades présentent des troubles du sommeil. Sur le plan thérapeutique l’évolution a été favorable chez 78,8% des patients sous AINS, Tramadol et Amitriptilline.

**Conclusion:**

otre travail à l'instar des études antérieures sur la NCB montre que cette pathologie reste une entité clinique relativement courante. Le pronostic généralement favorable est fonction d'un diagnostic précoce et d'une prise en charge adaptée.

## Introduction

La névralgie cervico-brachiale (NCB) est une douleur et / ou un syndrome caractérisé par un déficit sensorimoteur lié à une compression d'une racine cervicale [1]. Cette compression radiculaire est souvent liéé à un conflit disco-radiculaire dans le cadre d'une hernie discale, une arthrose, une spondylolystesis, une instabilité cervicale, un traumatisme ou rarement une tumeur [2, 3]. Cette pathologie reste une entité relativement fréquente dans la pratique courante et son incidence annuelle, ajustée est estimée à 83 pour 100.000 personnes. [1]. En outre il s'agit d'une pathologie de l'adulte en période d'activité professionnelle. Elle est par conséquent pourvoyeuse de douleur neurologique importante et handicapante, pouvant générer d'importants coûts médicaux, socio-économiques et un impact négatif sur la qualité de vie des patients [4]. Cependant, en dépit de son caractère très handicapant, cette pathologie reste très peu rapportée dans la littérature. Ainsi en France la revue de la littérature montre qu'elle est nettement moins étudiée que la névralgie sciatique [4]. En Afrique en général et au Mali en particulier, très peu d’études ont porté sur la NCB. Notre étude a donc pour objectif d’étudier les caractéristiques épidémiologiques et cliniques de la NCB et d'analyser les modalités thérapeutiques dans le contexte de notre exercice.

## Méthodes

### Design de l’étude et calcul de la taille de l’échantillon

Nous avons réalisé une étude longitudinale, prospective, descriptive et analytique qui s'est déroulée du 1er novembre 2009 au 30 Août 2010 en consultation dans le service de neurologie du CHU Gabriel Touré de Bamako. Nous avons inclus de manière consécutive l'ensemble des patients vus en consultation pour une NCB et dont le consentement a été obtenu.

### Recrutement des patients et considérations éthiques

Nous avons inclus, de manière consécutive les patients présentant cliniquement une NCB ayant les caractéristiques suivants: adultes âgés d'au moins 18 ans, patients vu en consultation de neurologie et présentant des capacités cognitives suffisantes pour comprendre le questionnaire. La traduction de notre questionnaire en bambara (langue nationale du pays) pour ceux de nos malades ne parlant pas français (langue officielle du pays) a été effectuée avec l'aide d'un expert linguistique en Bambara (DENAFLA).

Ont été exclus les patients ne signalant pas de NCB de manière nette, ceux présentant une autre cause évidente ou suspecte pouvant induire des douleurs de type neuropathique (diabète, cancer, malnutrition sévère, expositions à certains traitements comme les antituberculeux, autres pathologies infectieuses, métaboliques, intoxication à l'alcool). Ont été aussi exclus les patients au dossier incomplet et inexploitable, ceux présentant une malformation du rachis cervical et ceux vus en dehors de la période d’étude.

### Recueil des données cliniques et biologiques

Les patients inclus ont été examinés par les mêmes investigateurs cliniques (YM), initialement puis une fois par semaine au cours d'une consultation de routine, avec un interrogatoire suivi d'un examen neurologique et somatique général, et ce jusqu’à 2 mois après le diagnostic initial. Sur le plan de la douleur, il s'agit de patients présentant comme plainte, des douleurs cervico-brachiales uni ou bilatérales; systématisées ou non; avec ou sans déficit. Le caractère neuropathique de la douleur a été déterminé sur la base de la DN4 [5]. Les patients qui ayant un score supérieur ou égal 4/10 ont été inclus comme ayant une douleur neurologique. Pour l’évaluation de la douleur neurologique, une échelle catégorielle a été utilisée, l’échelle verbale simple (EVS). Il s'agit de demander verbalement au patient d'évaluer sa douleur selon 5 catégories permettant d'obtenir un score: pas de douleur, douleur faible, douleur modérée, douleur intense, douleur extrêmement forte [6, 7]. Pour l'impact de la douleur sur la qualité de vie, les échelles ont donc été les suivantes: l’échelle HAD (traduite en Bambara) [7]; le questionnaire concis sur les douleurs [8]. Un bilan biologique d'inclusion a été réalisé, comprenant: sérologie VIH, hémogramme, hépatique, ionogramme sanguin, sérologie syphilitique (TPHA/VDRL), calcémie, inflammation (VS, CRP), glycémie, créatinémie.

### Bilan radiographique

Une radiographie standard a été effectuée pour tous les patients, selon les modalités suivantes: incidence de face, de profil, de 3/4 et une étude dynamique en flexion et dégageant la charnière cervico-dorsale, la première côte et le dôme pleural. L'EMG, inaccessible, n'a pu être réalisé

### Traitement

Un protocole associant 3 molécules a été d'emblée mis en route à dose progressive: antidépresseur tricyclique (Amitriptyline), à raison de 25 mg comme dose d'attaque, à augmenter en fonction du résultat clinique (5 mg par semaine), avec une dose maximale moyenne de 75 mg par jour; antalgique de classe II (Tramadol), à raison de 50 mg par jour jusqu’à atteindre la dose maximale de 300 mg par jour; Kétoprofène: 300mg: 1cp. 2 /jour (pour les patients n'ayant pas d'ulcère gastrique documentée). Pour le suivi des malades nous avons utilisé une échelle catégorielle de soulagement [6]: aggravation de la douleur, pas de soulagement, soulagement faible, modéré, fort, complet.

### Collecte et analyse statistique des données

Les données ont été recueillies sur une fiche d'enquête individuelle comportant les paramètres socio démographiques (âge, sexe), les antécédents médicaux, les signes cliniques, para-cliniques et l’évolution des patients. La taille minimale de notre échantillon à été estimée à 52 individus, taille minimale calculée selon la formule de Kish [9], en utilisant une prévalence de 3,19% (données du service de Rhumatologie CHU Point G de Bamako sur la NCB, thèse de doctorat en médecine) [10], et un intervalle de confiance de 95%. La saisie et l'analyse statistique des données ont été réalisées avec le logiciel SPSS (version 16.0 Chicago, IL; USA). Des tableaux de fréquence et des calculs de moyennes ont été effectués. Le test de khi2 a été utilisé pour la comparaison des proportions avec un seuil de significativité fixé à p ≤ 0,05.

## Résultats

### Profil sociodémographique des patients neuropathiques

Sur la période de l’étude, nous avons reçu 477 patients en consultation et avons diagnostiqué 52 cas de NCB, soit une fréquence de 10,9%. Le sexe féminin est le plus touché (61,5%), avec un sexe ratio de 1,6. La classe modale est représentée par la tranche d’âge de 50-59 ans soit 21 cas (40,4%). L’âge moyen est de 48 ± 7 ans avec des extrêmes de 20 et 80 ans. Les ménagères sont les plus touchées, 21(40,4%). Les patients droitiers prédominent (94,2%; P = 0,0006).

### Profil clinique des patients neuropathiques

La cervicalgie est le principal signe fonctionnel soit 24 patients (46,2%). En ce qui concerne la durée d’évolution, 19 patients (36,5%) malades ont été vus un mois après l'apparition des premiers signes. La majorité des patients 36 (69,2%) n'a pas signalé de facteur déclenchant, mais seulement pour 7 patients (13, 5%) un contexte d'efforts inhabituels.

L'examen physique a noté une douleur à la mobilisation du rachis cervical chez 88,5% des patients. Pour la topographie de l'atteinte radiculaire, elle concerne respectivement la racine C7 pour 26 d'entre eux (50,0%), la C6 pour 12 (23, 1%), la C8 pour 7 (13, 5%). Quarante cinq patients, (86,53%) avaient un déficit sensitivomoteur correspondant à la topographie de la racine atteinte. La névralgie a été décrite comme intense chez 30 de nos patients (57,7%), quand au type de la douleur, la majorité a signalé des décharges électriques (25 patients = 48,1%). La souffrance radiculaire C1-C2 est apparue la plus douloureuse; cette différence n’étant pas statistiquement significative (P = 0,111). Le Tableau 1 résume les caractéristiques cliniques de la douleur.

**Tableau 1:** Caractéristiques cliniques de la névralgie cervico-brachiale

	Effectifs	Pourcentage %
**Signes fonctionnels**		
Cervicalgie	24	46,2
Douleur de l’épaule	4	7,7
Douleur du bras	4	7,7
Douleur de l'avant-bras	3	5,8
Douleur de la main	2	3,8
Douleur de l'ensemble du membre supérieur	15	28,8
**Durée des symptômes**		
Inférieur à 30 jours	11	21,2
Supérieur à 30 jours	19	36,5
Non précisé.	22	42,3
**Facteurs déclenchant**		
Effort inhabituel	7	13,5
Activité inhabituelles	9	17,3
Aucun	36	69,2
**Horaire de survenue de la douleur**		
Nocturne	39	75
Diurne	5	9,6
Permanent.	8	15,4
**Type de douleur selon la DN4**		
Décharges électriques	25	48,1
Crampes	10	19,2
Brûlure	3	5,8
fourmillement	9	17,3
Broiement	5	9,6
**Intensité de la douleur selon EVS**		
Douleur faible (EVS 1)	6	11,5
Douleur modérée (EVS 2)	11	21,2
Douleur intense (EVS 3)	30	57,7
Douleur très intense (EVS4)	5	9, 6

### Les données para cliniques

Le bilan biologique nous a permis de noter un syndrome inflammatoire biologique avec CRP supérieure à 10mg (taux normal < à 10) chez la majorité de nos patients (69,2%); cette différence est statistiquement significative (P = 0,002). Sur le plan radiologique, la quasi-totalité de nos patients a une radiographie anormale avec des signes d'arthrose évoluée. Le Tableau 2 résume les anomalies radiologiques retrouvées chez nos patients.

**Tableau 2:** Aspects radiologiques de la névralgie cervico-brachiale

Signes radiologiques	Effectifs	Pourcentage
Ostéophyte et pincement discal	18	34,6
Uncodiscarthrose et ostéophytes	20	38,5
Foramen rétrécis +uncoarthrose et ostéophytoses	7	13,5
Cervicarthrose étagée avec foramen normal	6	11,5
Pas d'anomalies	1	1,9
**Total**	**52**	**100,0**

### Impact de la douleur sur la qualité de vie des patients

**Figure 1:**
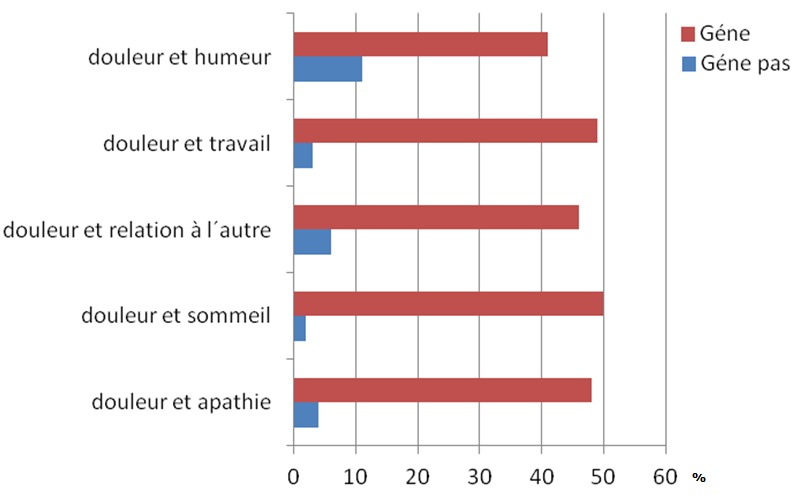
Impact de la douleur sur la qualité de vie des patients

La Figure 1 résume le retentissement de la douleur sur la qualité de vie des patients (humeur, capacité à la marche, travail habituel, sommeil, relation à autrui, goût de vivre. Globalement la NCB a un impact négatif sur la qualité de vie de la totalité des patients. Ce retentissement semble plus important sur la qualité du sommeil et sur le travail. La présence d'une anxiété selon l’échelle HAD est manifeste chez 90% des patients avec un score supérieur à 15, l'anxiété apparaît douteuse pour 8% avec un score inférieure à 10; seulement 2% des patients ne présentent pas d'anxiété avec un score inférieur à 7. La dépression selon l’échelle HAD est certaine pour 40% avec un score supérieur à 12 elle est douteuse (incertaine), avec un score inférieur à 10 pour 30%, et 30% n'ont pas de note dépressive.

### Aspects thérapeutiques

La majorité des patients a eu recours à un traitement antérieur avant notre consultation. 23 patients (44%) ont procédé à une automédication à base d'antalgique et de vitaminothérapie. 19 patients (36%) ont eu recours à un thérapeute traditionnel. Seulement 10 patients (19,2%) étaient à leur première consultation. Après un mois de traitement avec un protocole associant: immobilisation du rachis, amitriptilline, tramadol, Kétoprofene, nous avons observé la situation suivante: soulagement complet (26 patients = 50%); soulagement significatif (11 patients = 21, 15%); soulagement modéré (8 patients, 15, 38%); soulagement faible (4 patients, 7, 69%); pas de changement (2 patients, 3,84%); aggravation (1 patient, 1,92%)

## Discussion

Cette étude a donc visé à déterminer la prévalence, l'intensité, les facteurs associés, et l'impact de la NBC en consultation dans un service de neurologie au CHU de Bamako. L'incidence observée est nettement plus élevée que la moyenne retrouvée dans la littérature. Une étude rétrospective antérieure dans le service de rhumatologie du même CHU indiquait une incidence de 3.19% [10]. Cette discordance est sans doute liée aux critères d'inclusion des patients et aux profils des patients (étude hospitalière, patients en ambulatoires, outil diagnostic) et surtout au caractère rétrospective de l’étude avec des biais de sélection. L'utilisation d'outil fiable et validé pour le diagnostic de cette douleur neuropathique d'une part et d'autre le caractère prospectif de notre étude peut expliquer en partie l'incidence élevée de la NCB. Cependant il faut noter que cette névralgie reste une pathologie beaucoup moins fréquente que la sciatique [4].

Nous avons montré au cours de ce travail la prédominance de la NCB chez l'adulte en période d'activité professionnelle avec comme conséquence un impact socioprofessionnel et économique important. Nos résultats sont concordants avec les données de la littérature, par exemple au Togo, pays se situant dans la même sous région que le Mali [11]. En France, il a été rapporté des moyennes d’âge similaires avec cependant un âge de début précoce de la maladie, autour de 30 ans [4]. Nous avons noté aussi une prédominance féminine (61,23%) qui n'est pas systématiquement retrouvée dans la littérature; au Togo prédominance masculine, en France pas de différence significative entre les deux sexes [4]. Dans notre cas nous pensons que la prédominance féminine pourrait s'expliquer par une spécificité locale, inhérent aux activités ménagères des femmes. Au Mali culturellement, les femmes portent souvent des lourdes charges sur la tête. Cette hyper sollicitation du rachis cervical pourrait expliquer l'importance de la pathologie rachidienne cervicale chez les femmes ménagères. Une étude à plus grande échelle nous permettrait d'expliquer cette situation et surtout de faire un lien de causalité.

La douleur rachidienne cervicale est le signe fonctionnel inaugural, présent chez la majorité des patients. Ce constat est largement retrouvé dans la littérature [1, 12]. Elle serait en rapport avec les lésions inflammatoires sous jacentes. Nous avons noté effectivement un syndrome inflammatoire biologique chez la majorité des patients [12].

Sur le plan diagnostic, notre approche a été purement clinique (interrogatoire, examen physique, examen neurologique) avec la DN4 pour affirmer le caractère neurologique de la douleur. Cette approche est conforme aux données de la littérature: il est admis que l'histoire de la maladie à elle seule permet de confirmer75% de NCB avec une douleur cervicale associée à une douleur brachiale et une distribution en dermatome radiculaire spécifique d'une racine cervicale [13]. Dans notre étude la souffrance radiculaire porte essentiellement sur la racine C7; celle de Bouvier (50 patients) identifie 50% des patients sur cette racine [4]. Les causes de cette atteinte préférentielle de la C7 ne sont pas bien connues.

Sur le plan radiologique, l'arthrose cervicale évoluée chez la quasi-totalité de nos patients est connue comme étant la principale cause de la NCB [1, 4, 12]. L'impact très négatif de cette névralgie sur la qualité de vie des patients est rapporté par la majorité des auteurs [1, 4].

Sur le plan thérapeutique, l'utilisation de l'association Tramadol et antidépresseur tricyclique dans la prise en charge de la douleur neuropathique est bien validé avec un niveau d’évidence de grade A [14, 15]. L'intérêt d'une immobilisation du rachis cervical à l'aide de minerve est admis avec un niveau d’évidence de grade C [16]. Quand aux AINS, compte tenu du caractère inflammatoire de la NCB, ils sont considérés comme médicaments de première ligne par plusieurs auteurs [12].

Nous avons noté une évolution rapidement favorable chez la grande majorité de nos patients, ceci est conforme aux données de la littérature. La NCB, en dépit de son caractère aiguë et handicapant reste une pathologie bénigne dans sa forme classique quand elle est bien pris en charge [1, 4]. Ainsi, une étude épidémiologique à grande échelle au USA à montré que 90% des patients suivi pour NCB ont eu une évolution très favorable et étaient asymptomatiques après 5 ans de suivi [17]. Néanmoins, nous avons noté des cas de résistance au traitement, à savoir 8 patients avec soulagement modéré; 4 patients avec un niveau de soulagement faible; 2 patient n'ont pas noté de changement au traitement et un patient a même signalé une aggravation. En pratique, devant une NCB rebelle au traitement, le clinicien doit reconsidérer le diagnostic, et évoquer l'hypothèse d'une NCB secondaire (tumorale), justifiant le recours aux examens d'imagerie moderne (TDM/ IRM) [18].

## Conclusion

Notre travail à l'instar des études antérieures sur la NCB montre que cette pathologie reste une entité clinique relativement courante. Le caractère handicapant de cette névralgie de l'adulte en période d'activité professionnelle, et son impact négatif sur la qualité de vie des patients impose aux cliniciens un diagnostic rapide et la mise en route d'un traitement approprié. Le pronostic généralement favorable est fonction d'un diagnostic précoce et d'une prise en charge adaptée.
